# Doublecortin-Like Kinase 1 Is Elevated Serologically in Pancreatic Ductal Adenocarcinoma and Widely Expressed on Circulating Tumor Cells

**DOI:** 10.1371/journal.pone.0118933

**Published:** 2015-02-27

**Authors:** Dongfeng Qu, Jeremy Johnson, Parthasarathy Chandrakesan, Nathaniel Weygant, Randal May, Nicole Aiello, Andrew Rhim, Lichao Zhao, Wei Zheng, Stanley Lightfoot, Shubham Pant, Jeremy Irvan, Russell Postier, James Hocker, Jay S. Hanas, Naushad Ali, Sripathi M. Sureban, Guangyu An, Michael J. Schlosser, Ben Stanger, Courtney W. Houchen

**Affiliations:** 1 Department of Internal Medicine, University of Oklahoma Health Sciences Center, Oklahoma City, OK, United States of America; 2 Department of Surgery, University of Oklahoma Health Sciences Center, Oklahoma City, OK, United States of America; 3 Department of Pathology, University of Oklahoma Health Sciences Center, Oklahoma City, OK, United States of America; 4 Department of Biochemistry and Molecular Biology, University of Oklahoma Health Sciences Center, Oklahoma City, OK, United States of America; 5 Department of Veterans Affairs Medical Center, Oklahoma City, OK, United States of America; 6 Peggy and Charles Stephenson Oklahoma Cancer Center, Oklahoma City, OK, United States of America; 7 COARE Biotechnology Inc., Oklahoma City, OK, United States of America; 8 Department of Medicine, University of Pennsylvania, Philadelphia, PA, United States of America; 9 Department of Internal Medicine, University of Michigan, Ann Arbor, MI, United States of America; 10 Department of Oncology, Beijing Chaoyang Hospital, Capital Medicinal University, Beijing, China; Centro Nacional de Investigaciones Oncológicas (CNIO), SPAIN

## Abstract

Doublecortin-like kinase 1 (DCLK1) is a putative pancreatic stem cell marker and is upregulated in pancreatic cancer, colorectal cancer, and many other solid tumors. It marks tumor stem cells in mouse models of intestinal neoplasia. Here we sought to determine whether DCLK1 protein can be detected in the bloodstream and if its levels in archived serum samples could be quantitatively assessed in pancreatic cancer patients. DCLK1 specific ELISA, western blotting, and immunohistochemical analyses were used to determine expression levels in the serum and staining intensity in archived tumor tissues of pancreatic ductal adenocarcinoma (PDAC) patients and in pancreatic cancer mouse models. DCLK1 levels in the serum were elevated in early stages of PDAC (stages I and II) compared to healthy volunteers (normal controls). No differences were observed between stages III/IV and normal controls. In resected surgical tissues, DCLK1 expression intensity in the stromal cells was significantly higher than that observed in tumor epithelial cells. Circulating tumor cells were isolated from KPCY mice and approximately 52% of these cells were positive for Dclk1 staining. Dclk1 levels in the serum of KPC mice were also elevated. We have previously demonstrated that DCLK1 plays a potential role in regulating epithelial mesenchymal transition (EMT). Given the increasingly recognized role of EMT derived stem cells in cancer progression and metastasis, we hypothesize that DCLK1 may contribute to the metastatic process. Taken together, our results suggest that DCLK1 serum levels and DCLK1 positive circulating tumor cells should be further assessed for their potential diagnostic and prognostic significance.

## Introduction

Pancreatic ductal adenocarcinoma (PDAC) has the worst prognosis of any major malignancy with less than a 6% 5-year survival rate and is one of the leading causes of cancer-related death in the developed world. Even patients with resectable disease only have a five-year survival of <20%. At initial diagnosis, nearly 50% of the patients will have distant metastases in the liver or peritoneal surface, and more than 80% of those without distant spread will have locally advanced unresectable tumors [1]. PDAC tumors are considered highly aggressive with a fast progression rate and high metastatic potential. Extensive local tumor invasion and early metastasis complicate all current therapies for PDAC. Moreover, the heterogeneity of cell types within the tumor microenvironment and dense desmoplasia continue to be obstacles to conventional chemotherapy.

Cancer stem cells (CSCs) comprise < 5% of solid tumors, yet are capable of unrestrained self-renewal and are often resistant to traditional chemotherapy and radiation therapy treatments, as these cells do not rapidly divide [2]. The presence of CSCs was first established in acute myelogenous leukemia and later demonstrated in breast, pancreatic, and brain tumors [3–6]. A growing body of evidence suggests that stem cells may play a decisive role in the development and progression of cancer [7,8]. A cancer stem cell is defined as a cell within a tumor that is able to self-renew and to produce the heterogeneous lineages of cancer cells that comprise the tumor [8]. CSCs are often resistant to chemotherapy and radiation therapy, and this may explain why current treatments do not cure PDAC or prevent recurrence [2,9–12]. Recently, cells with CSC properties were identified in PDAC [5,13]. Li et al. identified a CD44^+^CD24^+^ESA^+^ population in PDAC which has a 100-fold increased tumorigenic potential compared to the rest of population [5]. Hermann et al reported that the CD133^+^CXCR4^+^ population in PDAC represents the CSC essential for tumor invasiveness and metastasis [13]. Invasive cancers are often characterized by epithelial-mesenchymal transition (EMT), a process in which immobile epithelial tumor cells can transform into highly metastatic and proliferative mesenchymal cells. EMT plays a key role in cancer invasion and metastasis [14,15]. PDAC cells that have undergone EMT have increased expression of the stem cell markers CD24, CD44, and ESA, and increased sphere-forming capacity, suggesting a link between EMT and CSCs in PDAC [12,16]. EMT in CSCs may play a critical role in tumorigenesis in general and PDAC in particular [17].

Doublecortin-like kinase 1 (DCLK1) is a microtubule-associated protein that has been identified as a tuft cell marker with stem-like properties in the pancreas [18–20]. DCLK1+ pancreatic cells represent less than 0.5% of the normal mouse pancreas, form spheroids in culture, and induce pancreatic epithelial expression when injected subcutaneously in nude mice [19]. DCLK1 is overexpressed in pancreatic cancer and correlated to pancreatic intraepithelial (PanIN) stage [21,22]. DCLK1+ cells are enriched in putative pancreatic CSC markers CD44, CD133, and CD24 [5,13,23,24]. In confirmation of DCLK1’s CSC status, recent studies indicate that DCLK1+ PDAC cells can initiate pancreatic tumorigenesis [22]. These data demonstrate that DCLK1 is a CSC marker and a potential therapeutic target for PDAC treatment.

In this report, we sought to determine whether DCLK1 protein can be detected in the bloodstream and if its levels in archived serum samples could be quantitatively assessed in pancreatic cancer patients and in PDAC mouse model. We also assessed the DCLK1 expression in stromal and epithelial compartments in pancreatic tumor tissues.

## Materials and Methods

### Ethics Statement

All participants in the study read an information sheet describing the risks, benefits and alternatives of participating in the study and provided informed consent. The study was approved by the University of Oklahoma Health Sciences Center (OUHSC) Institutional Review Board (16048).

### Specimen procurement

74 de-identified serum samples of normal subjects and patients with various stages of PDAC were obtained from Department of Surgery Research Core Lab OUHSC (n = 12, 12, 19, 16 and 15 for normal, stage I, II, III, and IV respectively). Among them, there are 37 females (mean age = 64, median age = 65, 34 White and 3 African American) and 37 males (mean age = 63, median age = 62, 35 White, 1 African American, and 1 Asian American). Stage-wise information is listed in Table 1. Pancreatic diseases were ruled out in the normal control group at the time of blood drawing. Forty-four banked paraffin-embedded pancreatic tumor tissues were also obtained from Department of Surgery Research Core Lab.

**Table 1:** General information of human subjects used in this study.

	Female	Male	Overall
**Normal Control**			
Sample Size	10	2	12
Mean Age	37	34	36
Median Age	34	34	34
White	9	2	11
Black/African	1	0	1
Asian	0	0	0
**Stage I PDAC**			
Sample Size	5	7	12
Mean Age	61	53	57
Median Age	65	52	54
White	5	7	12
Black/African	0	0	0
Asian	0	0	0
% Metastatic	0	14.3%	8.3%
**Stage II PDAC**			
Sample Size	10	9	19
Mean Age	70	65	68
Median Age	70	67	68
White	10	9	19
Black/African	0	0	0
Asian	0	0	0
% Metastatic	10%	33.3%	21.1%
**Stage III PDAC**			
Sample Size	5	11	16
Mean Age	67	67	67
Median Age	73	64	65
White	4	11	15
Black/African	1	0	1
Asian	0	0	0
% Metastatic	20%	36.4%	31.3%
**Stage IV PDAC**			
Sample Size	7	8	15
Mean Age	56	63	59
Median Age	57	64	61
White	6	6	12
Black/African	1	1	2
Asian	0	1	1
%Metastatic	100%	100%	100%

### Experimental animals

The KRas^LSLG12D^p53^LSLR172H^Pdx1^Cre^Rosa26YFP (KPCY) and control mice were described previously [25]. Mice were housed under controlled conditions, including a 12-h light-dark cycle, with ad libitum access to food and water. All animal experiments were performed with the approval and authorization from the Institutional Review Board and the Institutional Animal Care and Use Committee of the University of Pennsylvania and the University of Oklahoma Health Sciences Center.

### Mouse samples

Part of serum samples from 5- and 25-week old control and KPC mice (n = 3 for 25-week old KPC, n = 2 for other groups, both female and male) were generously provided by Dr. Surinder Batra of University of Nebraska Medical School. The rest of serum samples were collected from 5-, 15-, and 25-week old control and KPCY mice (n = 3 for 5- and 25-week old, and n = 5 for 15-week old, both female and male). Whole blood was also collected from three 16-week old KPCY mice (both female and male). Slides (n = 3 for each time interval) with fixed and processed pancreatic tissues of the C57Bl/6 mice treated with caerulein (50 μg/kg bodyweight, *i*.*p*. eight hourly injections per day for two days to induce acute pancreatitis [26]) were kindly provided by Drs. Maximilian Reichert and Anil Rustgi of the University of Pennsylvania.

### ELISA analysis

The serum DCLK1 level was quantified using a commercially available ELISA assay (USCN Life Science Inc., Wuhan, China). The 96-well plate coated with monoclonal antibody against DCLK1 was pre-blocked. Purified DCLK1 protein at different concentrations (0–10 ng/ml) was used to create a standard curve. Serum samples were diluted 1:4 and 1:10 with PBS. The diluted serum samples along with the purified DCLK1 proteins were added into the pre-blocked 96-well plate and incubated for two hours at room temperature. The plate was then incubated with biotinylated polyclonal antibody against DCLK1 for one hour at room temperature. After three washes, the plate was then incubated with Streptavidin conjugated with horseradish peroxidase (HRP) for thirty minutes at room temperature. Finally, the plate was developed with HRP substrate for twenty minutes and terminated by adding stop solution. The value of OD 450 nm was measured using a microplate reader and the concentration of DCLK1 in serum samples was determined based on the standard curve constructed using purified DCLK1.

The serum CA19-9 level was also quantified using a commercially available ELISA assay (Immuno-Biological Laboratories, Inc., Minneapolis, MN) according to the manufacturer’s instruction.

### Western blotting

Serum samples of these pancreatic cancer patients and of KPC mice were also analysis by western blotting. Serum samples (20 μl) were purified using ProteoSpin Abundant Serum Protein Depletion kit purchased from Norgen Biotek (Ontario, Canada). The samples were then separated on a 7% SDS-PAGE gel and transferred to an Immobilon membrane. Following blocking, the membrane was probed overnight with primary antibody (anti-DCLK1 ab31704, Abcam, Cambridge, MA) and subsequently with secondary antibody conjugated with horseradish peroxide for one hour. The 82-kilodalton DCLK1 protein was detected using ECL Western Blotting detection reagents (Amersham-Pharmacia). Serum DCLK1 expression levels were quantified using GelQuant software.

### Immunohistochemistry (IHC)

Heat-induced epitope retrieval was performed on 4-μm formalin-fixed paraffin-embedded sections by utilizing a pressurized Decloaking Chamber (Biocare Medical, Concord, CA) in citrate buffer (pH 6.0) at 99°C for 18 min. For brightfield microscopy, slides exposed to peroxidase blocking solution prior to the addition of primary antibodies. After incubation with primary antibodies (anti-cytokeratin mAb and anti-DCLK1 pAb) overnight at 4°C, the slides were incubated in peroxidase-conjugated polymer (Promark Series-Biocare Medical, CA). Slides were then developed with Betazoid DAB or Bajoran Purple HRP chromogens (Biocare Medical). Slides were examined with a Nikon 80i microscope and DXM1200C camera for brightfield microscopy. To detect Dclk1 in both primary and metastatic tumor tissues of KPCY mice, following the incubation with anti-Dclk1 pAb, anti-rabbit secondary antibody conjugated with Alexa 547 was used. Fluorescent images were taken with PlanFluoro objectives, utilizing a CoolSnap ES2 camera (Photometrics, Tucson, AZ). Images were processed using NIS-Elements software (Nikon Instruments, Melville, NY).

### IHC Scoring

Scoring of the DCLK1 staining was performed by three pathologists blinded to any patient information. Scoring was based on two different parameters: 1) staining intensity and 2) amount of tissue stained. The epithelial and stromal components were scored separately. Staining intensity was measured and scored from 0–3: no staining = 0, weak staining = 1, moderate staining = 2 and strong staining = 3. Similarly, the amount of tissue involved was measured and scored from 0–4: no tissue involved (0%) = 0, <10% involved = 1, 10%–50% involved = 2, 51%–80% involved = 3 and >80% involved = 4. Finally, the intensity score was multiplied by the tissue involvement score to obtain the DCLK1 staining score (e.g. 3 x 4 = 12).

### Detection of circulating tumor cells in mouse whole blood

The whole blood (100 μl) obtained from KPCY mice (n = 3, 16-week old, both male and female) was incubated with anti-Dclk1 antibody conjugated with AlexaFluor-546, and then subjected to flow cytometry analysis. Blood was initially sorted and gated based on YFP, and YFP+ cells were subsequently sorted for Dclk1. Data was collected on FACS Calibur and analyzed in ModFit LT.

### Analysis of DCLK1 mRNA levels in the pancreatic tumor tissues

Gene microarray data containing DCLK1 gene expression levels in PDAC tumor and normal pancreas tissue was downloaded from the NCBI gene expression omnibus (GEO) Series GSE11838 [27] and analyzed.

### Statistical analysis

Data was analyzed using the Mann-Whitney *U* test for pairwise comparisons between normal healthy controls and each PDAC stage group. For multiple comparisons, one-way ANOVA was performed using Bonferroni’s multiple comparisons test for correction. A *P value <*0.05 was considered statistically significant. A receiver operating characteristic (ROC) curve was constructed to assess diagnostic properties. All statistical analyses were performed using Graphpad Prism 6.0.

## Results

### DCLK1 levels in the serum of stage I-II PDAC patients are higher than in normal controls

**Fig 1:**
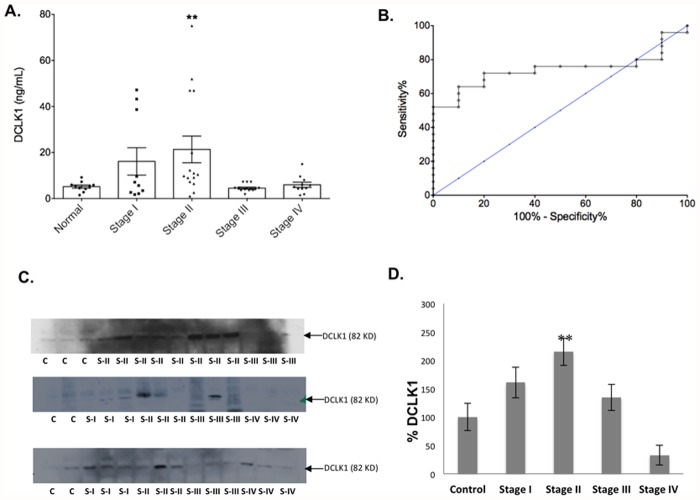
DCLK1 protein levels are elevated in the serum of stage I and II PDAC patients. A: DCLK1 protein levels in the serum samples were determined by ELISA. DCLK1 levels were grouped based on the cancer stage and compared to normal group. **p<0.05. n = 12, 12, 19, 16 and 15 for normal, stage I, II, III, and IV respectively. B: Receiver operating characteristic (ROC) curve analysis for the use of DCLK1 as serum marker for stage I and II PDAC (AUC = 0.74, and p = 0.029). C: DCLK1 protein levels in the serum samples were detected by western blotting. D: Percentage of DCLK1 levels in each stage relative to control based on western blotting analysis. **p<0.002.

To determine DCLK1 levels in the bloodstream, 74 de-identified serum samples of normal controls and patients with various stages of PDAC were used in a DCLK1 specific ELISA analysis. DCLK1 protein levels were determined, and were elevated in both stages I and II PDAC patients relative to controls (Fig. 1A). DCLK1 levels in the normal control, stage I, II, III, and IV groups were in the range of 1.50–9.19, 1.76–47.22, 0.74–75.12, 1.8–7.17, and 0.53–14.97 ng/ml, with median levels of 5.6, 6.2, 9, 4.1, and 5 ng/ml, respectively. The DCLK1 levels in the serum were significantly higher in stage II patients compared to normal control. Even with 4 highest values removed in stage II, the serum DCLK1 levels in stage II were still significantly higher than normal groups (p<0.03). Interestingly, DCLK1 levels in the serum of stage III and IV patients returned to levels similar to normal controls. Receiver operating characteristic (ROC) analysis was performed to evaluate the diagnostic utility of DCLK1 results of stages I and II patients (Fig. 1B). The *Area under the curve* (AUC) was 0.74, both sensitivity and specificity were significant (p = 0.029). DCLK1 protein levels were also detected using western blotting analysis, and were significantly higher in stage II patients compared to controls (Fig. 1C and D).

**Fig 2:**
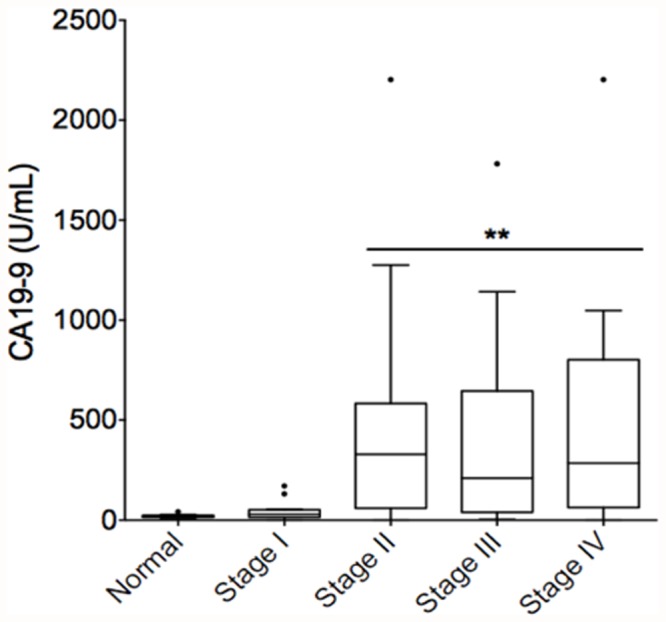
CA 19–9 levels are increased in the serum of stage II, III, and IV PDAC patients. CA 19–9 in the same serum samples was detected by ELISA. CA 19–9 levels were grouped based on the cancer stage and compared to normal group. **p<0.05. n = 12, 12, 19, 16, and 15 for normal, stage I, II, III, and IV respectively.

Carbohydrate antigen (CA) 19–9 is currently the only available serum marker for PDAC and has shown some utility as a diagnostic adjunct and a prognostic marker [28]. To compare the diagnostic accuracies of serum DCLK1 and CA19-9, the CA19-9 level in the same serum samples were also measured (Fig. 2). The CA19-9 levels in the serum were dramatically increased in stage II, III and IV patients. The ROC analysis indicated that the AUC was 0.85, both sensitivity and specificity were significant (p = 0.0001). There was no correlation between DCLK1 and CA19-9 values stage-wise.

### Distinct expression of Dclk1 is associated with acinar ductal metaplasia (ADM)

**Fig 3:**
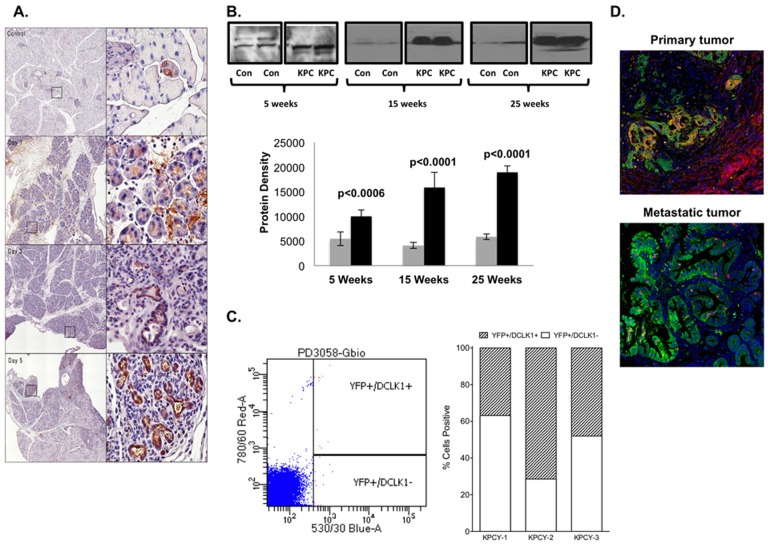
Dclk1 expression levels were evaluated in mouse models. A: Distinct expression of Dclk1 is associated with ADM. Dclk1 immunostaining in pancreas of C57Bl/6 mice treated with caerulein on day 0 (control), 1, 3, and 5 post injection. B: Dclk1 protein levels are upregulated in the serum of pre-cancerous and cancerous KPC mice. The serum of 5-, 15- (pre-cancerous) and 25- (cancerous) week old control (con) and KPC mice (n = 5 for each group) was purified and subjected to SDS-PAGE and immuno-blotted with anti-Dclk1 antibody. Dclk1 levels are upregulated in serum of pre-cancerous and cancerous KPC mice relative to controls (*p*<0.01). C: Dclk1 is expressed on the surface of circulating tumor cells in KPCY mice. Whole blood was collected from 4-month old KPCY mice (n = 3), incubated with anti-Dclk1 antibody conjugated with AlexaFluor-546, and subjected to flow cytometry. Among the YFP+ circulating cells, 52% were positive for Dclk1. D: Dclk1 is co-localized with YFP in both primary and metastatic tumors. Immuno-staining demonstrating both primary PDAC tissue and metastatic PDAC tissue (colon) of KPCY mice express Dclk1 (red) co-localized with YFP+ tumor cells (green). Dclk1/YFP double positive cells appear yellow-orange.

As there is no way to evaluate pre-neoplasia (i.e. PanIN lesions) in humans prospectively, we evaluated Dclk1 expression levels in animal models. ADM occurs during acute pancreatitis and may be considered a prelude to PanIN and PDAC development [29,30]. The fixed and processed pancreatic tissues of the C57Bl/6 mice treated with caerulein [26] were immuno-stained with anti-Dclk1 antibody (Fig. 3A). We found sparse to no staining of Dclk1 in normal pancreas with notable staining only in a single peri-acinar duct. Pancreatic tissue from day 1 following caerulein-induced injury was consistent with modest pancreatitis, and strong Dclk1 staining was observed in several small ducts. At day 3 following injury, characteristics consistent with severe pancreatitis were observed, and all ductules and ducts stained modestly with Dclk1 antibody. At day 5 following injury severe pancreatitis persisted, and all ductules and ducts stained strongly with Dclk1 antibody. These data demonstrate that Dclk1 is overexpressed in ductal cells following acute pancreatitis-induced ADM.

### Serum Dclk1 protein and Dclk1+ circulating tumor cells are present in mice with pre-neoplasia

The KPC mouse line is a well-established mouse line for pancreatic cancer model [25,31]. The KPC mice exhibit PanIN lesions as early as 8 weeks and tumor development as early as 16 weeks. Dclk1 protein expression in the serum of 5-, 15-, and 25- week old KPC mice are significantly elevated compared to control mice (Fig. 3B). Using the KPCY mouse model, Rhim et al. demonstrated that YFP+ circulating tumor cells can be isolated from the blood of KPCY mice as early as 8 weeks, indicating that EMT is a very early event in PDAC [25]. We found that approximately 52% of YFP+ cells in the blood of KPCY mice are Dclk1+ (Fig. 3C), suggesting that a large portion of circulating tumor cells are Dclk1+ cells. In addition, Dclk1 is co-localized with YFP in both primary and metastatic tumors of these KPCY mice (Fig. 3D). These results demonstrate that Dclk1 is a significant factor in the well-established KPC mouse model of PDAC.

### DCLK1 expression is higher in the stromal cells than that in epithelial cells

**Fig 4:**
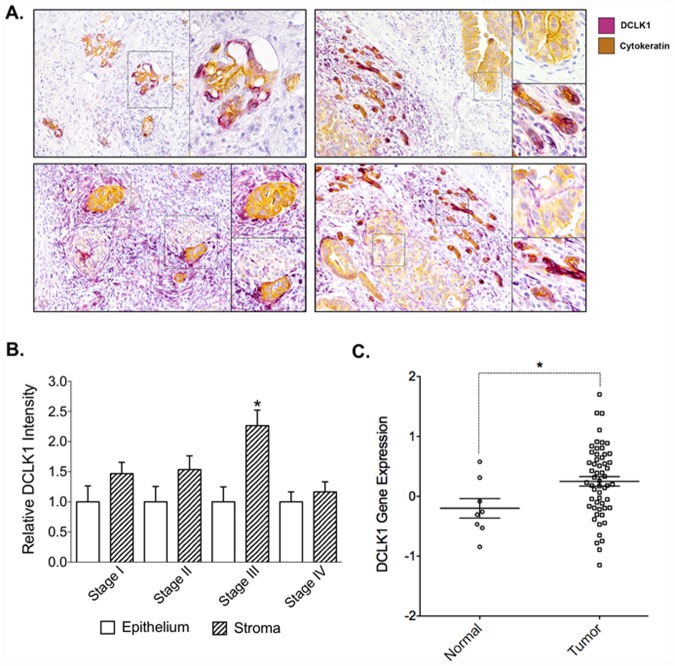
DCLK1 expression level is higher in stromal relative to epithelial cells in human PDAC. A: Paraffin-embedded PDAC tumor sections were immuno-stained with anti-cytokeratin antibody (yellow brown) and anti-DCLK1 antibody (purple) to detect tumor epithelial cells and DCLK1 expression. B: The intensity of DCLK1 expression is stronger in stromal relative to epithelial cells in human PDAC (p = 0.009). The intensity of DCLK1 expression in epithelial cells and stromal cells was scored separately for each specimen and grouped based on cancer stage (n = 10, 12, 12, and 10 for stage I, II, III, and IV respectively). C: DCLK1 mRNA levels are elevated in human pancreatic tumor tissue relative to normal pancreas tissue. DCLK1 mRNA expression levels were summarized and grouped using a published DNA microarray data [27]. p<0.05.

In our previous study using immunohistochemical (IHC) analysis, we demonstrated DCLK1 located in islets but not in the intervening stromal cells within the histologically normal appearing resection specimens [21]. DCLK1 however, was found in the islets, ducts and a few intervening stromal cells in chronic pancreatitis, and even more intense DCLK1 expression was found in ductal epithelial cells and in intervening stromal elements in pancreatic cancer [21]. We obtained 44 banked paraffin-embedded pancreatic tumor tissues and the AJCC cancer stage of these specimens was identified retrospectively. DCLK1 expression in these specimens was identified using IHC analysis. As reported previously, strong DCLK1 expression was found in ductal epithelial cells and intervening stromal elements in pancreatic cancer (Fig. 4A, purple staining). The epithelial cells in these tissues were also immuno-stained with anti-cytokeratin antibody (Fig. 4A, yellow brown staining). The intensity of DCLK1 staining in epithelial cells and stromal cells of each specimen was scored separately (Table 2). The intensity of DCLK1 expression in stromal elements was stronger than that in the tumor epithelial cells. Interestingly, a statistically significant increase was found in stage III tumors (Fig. 4B). Since stromal elements are mainly mesenchymal cells, these data suggest that DCLK1 expression is upregulated in both epithelial and mesenchymal cells of pancreatic tumor microenvironment.

**Table 2:** DCLK1 staining scores in epithelial and stromal cells of PDAC patients.

TNM Stage	Patient Identifier	Epithelial Scoring	Stromal scoring
I	3	1 x 3 = 3	2 x 3 = 6
I	45	1 x 3 = 3	3 x 3 = 9
I	47	3 x 4 = 12	3 x 4 = 12
I	48	3 x 2 = 6	3 x 4 = 12
I	51	1 x 1 = 1	2 x 3 = 6
I	52	3 x 4 = 12	3 x 4 = 12
I	53	3 x 4 = 12	3 x 4 = 12
I	54	1 x 2 = 2	2 x 3 = 6
I	55	3 x 4 = 12	3 x 4 = 12
I	57	0	1 x 3 = 3
**II**	14	1 x 2 = 2	2 x 3 = 6
**II**	28	1 x 1 = 1	1 x 3 = 3
**II**	29	3 x 1 = 3	2 x 3 = 6
**II**	30	1 x 1 = 1	2 x 3 = 6
**II**	34	3 x 4 = 12	3 x 4 = 12
**II**	35	3 x 4 = 12	3 x 4 = 12
**II**	39	1 x 3 = 3	1 x 3 = 3
**II**	40	1 x 3 = 3	1 x 3 = 3
**II**	44	3 x 2 = 6	3 x 3 = 9
**II**	46	3 x 2 = 6	3 x 3 = 9
**II**	56	1 x 1 = 1	2 x 3 = 6
**II**	58	3 x 4 = 12	3 x 4 = 12
**III**	1	3 x 2 = 6	3 x 3 = 9
**III**	2	1 x 3 = 3	3 x 4 = 12
**III**	31	1 x 3 = 3	1 x 3 = 3
**III**	32	1 x 3 = 3	3 x 4 = 12
**III**	33	2 x 3 = 6	3 x 4 = 12
**III**	36	1 x 2 = 2	2 x 3 = 6
**III**	37	3 x 4 = 12	3 x 4 = 12
**III**	38	0	3 x 2 = 6
**III**	41	2 x 1 = 2	2 x 3 = 6
**III**	42	2 x 1 = 2	3 x 4 = 12
**III**	43	3 x 4 = 12	3 x 4 = 12
**III**	49	3 x 2 = 6	3 x 2 = 6
**IV**	5	1 x 2 = 2	3 x 4 = 12
**IV**	8	1 x 1 = 1	1 x 3 = 3
**IV**	9	3 x 4 = 12	3 x 4 = 12
**IV**	18	3 x 2 = 6	2 x 3 = 6
**IV**	19	3 x 3 = 9	3 x 3 = 9
**IV**	23	3 x 4 = 12	3 x 4 = 12
**IV**	24	3 x 4 = 12	3 x 4 = 12
**IV**	25	3 x 4 = 12	3 x 4 = 12
**IV**	26	3 x 4 = 12	3 x 4 = 12
**IV**	27	2 x 2 = 4	2 x 4 = 8

DCLK1 mRNA levels in the tumor tissues are significantly higher than that in normal control. Balagurunathan et al. used gene expression profiling to identify cell-surface targets for multimeric ligands in pancreatic cancer [27]. Using their DNA microarray data, we analyzed DCLK1 gene expression levels in PDAC versus normal controls. DCLK1 mRNA levels were significantly higher in the tumor samples compared to normal controls (Fig. 4C). These data lend support to the observation that DCLK1 gene expression is upregulated in pancreatic cancer.

## Discussion

In this report we evaluated DCLK1 levels in the serum of PDAC patients and found that DCLK1 levels were significantly higher in stage I-II PDAC patients but fell precipitously back to normal in advanced stages. Serum Dclk1 levels were also increased in the pancreatic cancer KPC mice. In addition, more than 50% of circulating tumor cells isolated from the blood of KPC mice are DCLK1 positive. In human PDAC tissue, DCLK1 immunostaining indicated that DCLK1 expression was higher in the stromal cells than that in tumor epithelial cells, suggesting a potential conversion to mesenchymal cells during the early tumorigenic process.

In spite of enormous progress in understanding the molecular pathways associated with growth and metastasis of pancreatic cancer, definitive molecular marker(s) associated with cancer pathogenesis and prognosis has not been found. Currently, only CA19-9 has been used as a clinical biomarker to follow patient response for the treatment of pancreatic cancer and predicts overall and disease-free survival [32]. The data presented here demonstrated that DCLK1 serum levels are elevated in PDAC stages I and II, a potentially clinical relevant finding [33]. Although CA19-9 serum levels were better correlated with overall stage-wise tumor progression compared to DCLK1, further studies will be needed to assess whether increased serum expression of DCLK1 in stage I and II PDAC is a relevant predictor of metastatic progression, surgical and chemotherapeutic response, and patient survival. A recent report by Rhim et al. suggested that EMT in PDAC occurs much earlier than previously thought [25]. They were able to detect cells undergoing EMT using a novel KPCY mouse model where YFP labeled pancreatic epithelial cells were transformed into mesenchymal cells and were detectable very early in tissues and in the bloodstream. Several of these rare cells were identifiable in micrometastases. More than 50% of circulating YFP+ tumor cells are positive for Dclk1, suggesting that DCLK1+ tumor cells are among the cells undergoing EMT. We have previously reported that DCLK1 is upregulated in pancreatic tumor tissues and co-localizes with vimentin, a marker of mesenchymal lineage within premalignant PanIN lesions, suggesting that these DCLK1+ cells are of mesenchymal origin or in the process of EMT [21]. We found that downregulation/inhibition of DCLK1 by specific siRNA (siDCLK1) or kinase inhibitors results in downregulation of EMT associated factors and tumorigenesis [21,34–36]. These data suggest that DCLK1 may regulate EMT and tumorigenesis.

Our findings of increased Dclk1 expression in the ADM and pancreatitis in C57Bl/6 mice are consistent with recent reports in early stage KC^iMist1^ and KC^Pdx1^ mice, and *Ptf1a* ^Cre/+^;SOX17^OE^ mice [22,37]. We have also previously found DCLK1 expression is significantly higher in human PanIN lesions relative to normal ducts [21]. The composite scoring of DCLK1 expression suggests a stage-dependent increase in PanIN I to III compared to normal ductal epithelia [21]. Bailey et al. have also reported that pancreatic Dclk1+ cells are significantly increased in ADM, PanIN, and invasive PDAC relative to normal pancreatic ductal epithelium in KPC mice [22].

A recent report by Nakanishi et al. using a compound mouse line (Dclk1-Cre-ERT;*Apc* ^*min/+*^) confirmed that Dclk1 selectively marks tumor stem cells in the *Apc* ^*Min/+*^ mice, and ablation of Dclk1+ cells results in a marked regression of polyps without apparent damage to the normal mouse intestine [23]. These data suggest that Dclk1 can be used to distinguish between normal and tumor stem cells, a fundamental feature required for any potential therapeutic agent. Recent studies using multiple mouse models of PDAC strongly suggest that the Dclk1+ pancreatic tuft cell plays an equivalent role in pancreatic tumorigenesis [22,37]. Further studies utilizing Dclk1 lineage tracing mice as well as in-depth analysis of human PDAC patients will be needed to unequivocally confirm these findings. If these findings are confirmed, the development of targeted agents against DCLK1+ tumor stem cells may inhibit PDAC progression and metastasis and ultimately extend patient survival.
